# Rice bran derivatives alleviate microglia activation: possible involvement of MAPK pathway

**DOI:** 10.1186/s12974-016-0615-6

**Published:** 2016-06-14

**Authors:** Harsharan S. Bhatia, Julian Baron, Stephanie Hagl, Gunter P. Eckert, Bernd L. Fiebich

**Affiliations:** Department of Psychiatry, University of Freiburg Medical School, Hauptstr. 5, Freiburg, 79104 Germany; Department of Pharmacology, Goethe University, Biozentrum Niederursel, Max-von-Laue-Str. 9, Frankfurt, 60438 Germany; VivaCell Biotechnology GmbH, Ferdinand-Porsche-Str. 5, Denzlingen, 79211 Germany; Institute of Nutritional Sciences, University of Giessen, Wilhelmstrasse 20, Giessen, 35392 Germany

**Keywords:** Microglia activation, Neurodegeneration, Rice bran extract, Prostaglandins, Cytokines, Isoprostane

## Abstract

**Background:**

Hyperactivation of microglia is considered to be a key hallmark of brain inflammation and plays a critical role in regulating neuroinflammatory events. Neuroinflammatory responses in microglia represent one of the major risk factors for various neurodegenerative diseases. One of the strategies to protect the brain and slow down the progression of these neurodegenerative diseases is by consuming diet enriched in anti-oxidants and polyphenols. Therefore, the present study aimed to evaluate the anti-inflammatory effects of rice bran extract (RBE), one of the rich sources of vitamin E forms (tocopherols and tocotrienols) and gamma-oryzanols, in primary rat microglia.

**Methods:**

The vitamin E profile of the RBE was quantified by high-performance liquid chromatography (HPLC). Microglia were stimulated with lipopolysaccharide (LPS) in the presence or absence of RBE. Release of prostaglandins (prostaglandin (PG) E_2_, 8-iso-prostaglandin F_2α_ (8-iso-PGF_2α_)) were determined with enzyme immunoassay (EIA). Protein levels and genes related to PGE_2_ synthesis (Cyclooxygenase-2 (COX-2), microsomal prostaglandin E synthase-1 (mPGES-1)) and various pro- and anti-inflammatory cytokines (TNF-α, IL-1β, IL-6, and IL-10), were assessed by western blot, ELISA, and quantitative real-time PCR. Furthermore, to elucidate the molecular targets of RBE, the phosphorylated state of various mitogen-activated protein kinase (MAPK) signaling molecules (p38 MAPK, ERK 1/2, and JNK) and activation of NF-kB pathway was studied.

**Results:**

RBE significantly inhibited the release of PGE_2_ and free radical formation (8-iso-PGF_2α_) in LPS-activated primary microglia. Inhibition of PGE_2_ by RBE was dependent on reduced COX-2 and mPGES-1 immunoreactivity in microglia. Interestingly, treatment of activated microglia with RBE further enhanced the gene expression of the microglial M2 marker IL-10 and reduced the expression of pro-inflammatory M1 markers (TNF-α, IL-1β). Further mechanistic studies showed that RBE inhibits microglial activation by interfering with important steps of MAPK signaling pathway. Additionally, microglia activation with LPS leads to IkB-α degradation which was not affected by the pre-treatment of RBE.

**Conclusions:**

Taken together, our data demonstrate that RBE is able to affect microglial activation by interfering in important inflammatory pathway. These in vitro findings further demonstrate the potential value of RBE as a nutraceutical for the prevention of microglial dysfunction related to neuroinflammatory diseases, including Alzheimer’s disease.

**Electronic supplementary material:**

The online version of this article (doi:10.1186/s12974-016-0615-6) contains supplementary material, which is available to authorized users.

## Background

Neuroinflammation is one of the critical events in the progression of various neurodegenerative disorders such as multiple sclerosis (MS), Parkinson’s disease (PD), amyotrophic lateral sclerosis (ALS), and Alzheimer’s disease (AD) [1–4]. The main cell types responsible for the enhanced inflammation in various CNS pathologies are glial cells. Among them, activated microglia are principally involved in the maintenance and progression of neuroinflammation [5]. Microglia are resident immune cells in CNS and regarded as the primary component of the brain immune system. Under physiological conditions, these cells are constantly engaged in scanning their microenvironment for any exogenous or endogenous signals in efforts to maintain the homeostasis [6]. Infection, traumatic injury, ischemia, neurodegenerative diseases, or any altered neuronal activity indicating a potential threat to CNS can evoke profound changes in the microglial morphology and function [5, 7–9]. Under activated state, microglia is known to release a variety of cytotoxic mediators such as reactive oxygen species (ROS), pro-inflammatory cytokines, adenosine triphosphate (ATP), and arachidonic acid (AA) derivatives [10–13]. These over-released mediators further exert their toxic effects on the healthy neurons and result in a vicious and self-propagating cycle of neuronal death [14]. Therefore, microglial activation-associated inflammation serves as an important model for investigating potential therapeutic entities for slowing the progression of neuronal cell death in neurodegenerative disorders.

Prostaglandin E_2_ (PGE_2_) is AA derivative, the production of which is catalyzed by the cyclooxygenases and prostaglandin (PG) E synthases (PGESs) enzymes. Cyclooxygenases exist in two subtypes, cyclooxygenase-1 (COX-1) and cyclooxygenase-2 (COX-2). COX-2 has been shown to be overexpressed by bacterial cell wall component lipopolysaccharide (LPS) in cultured microglia [15]. The final step in the synthesis of PGE_2_ is regulated by PGESs. To date, three PGESs have been characterized: the microsomal PGESs (microsomal prostaglandin E synthase-1 (mPGES-1) and mPGES-2) and the cytosolic PGES (cPGES) [16, 17]. Among these, mPGES-1 is an inducible enzyme, and it has been shown to be upregulated in activated microglia [18]. These enzymes are regulated by a variety of intracellular signaling molecules such as nuclear factor-kappa B (NF-kB) and mitogen-activated protein kinases (MAPK). NF-kB is a transcription factor, and it has been shown to be a central regulator of inflammatory response. MAPKs are a family of serine/threonine protein kinases which are critical for the production of inflammatory mediators. Some of the main kinases in this group include extracellular signal-regulated kinases (ERK 1/2), c-Jun N-terminal kinase (JNK) and p38 isoforms. In particular, increased activity of p38 MAPK has been closely associated with pathologies involved in neurodegenerative diseases [19]. Several studies have demonstrated that p38 MAPK is upregulated during microglial inflammation [20]. Evidence suggests that patients with neurodegenerative diseases such as AD might benefit from p38 MAPK inhibitors [21]. Therefore, there is a need to develop compounds which can interfere with the activities of these kinases, hence modulate age related as well as activated microglia-associated neuroinflammatory diseases.

Recently, it has been revealed that natural components, plant extracts, and polyunsaturated fatty acids are useful for the prevention of neurodegenerative diseases [22, 23]. Extracts containing polyphenols and vitamin E have shown to confer neuroprotection in animal models [24, 25]. One of such extracts containing these components is rice bran extract (RBE). Rice bran is obtained as a by-product in the rice milling process, a method in which the outer layer of rice grain is removed. Rice bran and its ingredients have been shown to impart a number of health-promoting functions. These functions include cholesterol-lowering, prevention of ulcer formation, anti-oxidant, anti-inflammatory, anti-diabetic, and immunomodulatory effects [26–29]. The main components of rice bran are tocotrienols, tocopherols, and oryzanols (a mixture of ferulic acid esters of triterpene alcohol and phytosterols) [30]. In comparison with pure rice bran (which gets quickly rancid, leaving bran inedible), the above mentioned components are enriched with stabilized Egyptian rice bran extract. The recent studies are showing the beneficial effects of stabilized Egyptian RBE in aging- and dementia-associated disease models. For instance, Hagl et al. reported that the oral consumption of RBE for 3 weeks improved aging-related mitochondrial dysfunction in 18-month-old Naval Medical Research Institute mice (NMRI) [31]. In an another attempt, authors showed that RBE compensates mitochondrial dysfunction in an in vitro model of early AD [32]. More recently, RBE-supplemented diet showed its potential in decreasing inflammation associated to metabolic disorders [33]. To date, it is not established whether RBE interferes in microglial activation, a critical event in inflammation-associated neurodegenerative diseases. Consequently, we have investigated the effects of RBE in LPS-activated primary rat microglia.

## Methods

All experiments and animal procedures were performed according to the guidelines of ethics committee of University of Freiburg Medical School under approved protocol (Nr. X-13/06A). Animals were obtained from Center for Experimental Models and Transgenic Services-Freiburg (CEMT-FR). Maximum efforts were made to minimize the number of animals used and their suffering during this study.

### Chemicals

Heat-stabilized Egyptian rice bran extract was kindly provided by Dr. Amr Helal from IT&M S.A. (Giza, Egypt). After overnight maceration in ethanol, three successive extraction sessions under reflux at 40 °C were applied. The extraction ratio was 3:1. The extract was evaporated under vacuum at a temperature not exceeding 50 °C. The vitamin E profile of the RBE was quantified by HPLC, described elsewhere [30, 34] and as follows (all values in μg/g): alpha-tocopherol, 86; beta-tocopherol, 71; gamma-tocopherol, 288; delta-tocopherol, 93; alpha-tocotrienol, 55; beta-tocotrienol, not detectable; gamma-tocotrienol, 2226; and delta-tocotrienol, 266. LPS from *Salmonella typhimurium* (Sigma Aldrich, Deissenhofen, Germany) was resuspended in sterile phosphate-buffered saline (PBS, 5 mg/ml) as stock, subsequently used at a final concentration (10 ng/ml) in the cultures. The (±)-α-tocopherol (Sigma Aldrich, Deissenhofen, Germany) was dissolved in ethanol as 100 mM stock solution. Recombinant macrophage-colony stimulating factor (M-CSF) was used at 50 ng/ml as final concentration (Thermo Fisher Scientific, Darmstadt, Germany).

### Primary microglia cultures

Primary mixed glial cell cultures were established from cerebral cortices of 1-day neonatal Sprague–Dawley rats as described in our previous studies [35]. In brief, brains were carefully taken. Cerebral cortices were collected and freed from meninges. Forebrains were then minced and gently dissociated by repeated pipetting in Dulbecco’s modified Eagle’s medium (DMEM) and filtered by passing through 70-μm nylon cell strainer (BD biosciences, Heidelberg, Germany). Cells were collected by centrifugation (1000*g*, 10 min) and resuspended in DMEM containing 10 % fetal calf serum (FCS) (GE Healthcare, Germany) and antibiotics 1 % penicillin and streptomycin (40 U/ml and 40 μg/ml, respectively) (Sigma Aldrich, Germany). Cells were then cultured on 10-cm cell culture dishes (Falcon, Heidelberg, Germany) with the density of 5 × 10^5^ cells/ml in 5 % CO_2_ at 37 °C. After 12–14 days in vitro, floating microglia were harvested from mixed glia (astrocyte-microglia) cultures and re-seeded into cell culture plates at the density of either 2 × 10^5^ cells/well or 1.2 × 10^6^ cells/dish. On the next day, medium was removed to get rid of non-adherent cells and fresh medium was added. After 1 h, the cells were stimulated for respective experiments.

### Cell viability assay

Viability of primary rat microglia after treatment with RBE was measured by the CellTiter-Glo® Luminescent Cell Viability Assay (Promega). This assay is used to determine the number of metabolically active and viable cells, based on the quantitation of ATP present in the cells. Cells (2 × 10^5^/ml) were cultured for 24 h, subsequently incubated with RBE (10–300 μg/ml) for 24 h. Thereafter, the cells were treated with or without LPS (10 ng/ml) for the next 24 h. RBE was dissolved in ethanol and ethanol was used in the control wells (at final conc. of 0.15 %) during experiments. After 48 h of incubation, the concentration of ATP was measured after adding 100 μl of reconstituted substrate (for 10 min). Thereafter, luminescence was measured in GloMax® Luminometer (Promega).

### Determination of PGE_2_ and 8-iso-PGF_2α_ production from LPS-activated microglia

Cultured primary rat microglia were incubated with either RBE (10–300 μg/ml) or with α-tocopherol (10–100 μM) for 24 h. Afterwards, the cells were treated with or without LPS (10 ng/mL) for the next 24 h. After the end of the incubation period, supernatants were collected and centrifuged at 1000*g* for 5 min at 4 °C. PGE_2_ and 8-iso-prostaglandin F_2α_ (8-iso-PGF_2α_) production was assessed in cell supernatants with a commercially available enzyme immunoassay (EIA) kit (Biotrend, Cologne, Germany, and Cayman Chemicals, Ann Arbor, Michigan, USA) respectively, followed by measurement at 450 nm according to manufacturer’s instructions. For PGE_2_, standards from 39 to 2500 pg/ml were used and sensitivity of the assay was 36.2 pg/ml. The 8-iso-PGF_2α_ assay has a range of standards from 0.5 to 500 pg/ml and sensitivity approximately of 3 pg/ml.

### Determination of TNF-α, IL-1, and IL-6 release in microglia

Effects of RBE and α-tocopherol were also studied by determining the release of various cytokines. Briefly, microglia were pre-incubated either with RBE (50–300 μg/ml) or with α-tocopherol (10–100 μM). Afterwards, LPS (10 ng/ml) was added for 24 h, and release of tumor necrosis factor (TNF)-α and IL-6 was determined in the cell supernatants which were collected after centrifugation at 1000*g* for 5 min at 4 °C. For the release of IL-1β at the end of the incubation, ATP (1 mM) was added for 30 min in all the wells followed by the collection of cell supernatants. Addition of ATP was important since maturation and release of IL-1β is dependent on the IL-1β converting enzyme (ICE)/caspase 1. For determination of TNF-α (eBioscience, Frankfurt, Germany), IL-6 (Thermo Fisher Scientific, Darmstadt, Germany) and IL-1β (R&D Systems Europe, Ltd., Abingdon, UK), commercially available ELISA kits were used. All the measurements were done at 450 nm according to the manufacturer’s instructions. For TNF-α, standards from 16 to 2000 pg/ml were used with sensitivity 16 pg/ml. IL-6 has a range between 23.5 and 1500 pg⁄ml with sensitivity <5 pg/ml, and IL-1β standards were between 31.5 and 2000 pg/ml with sensitivity of <5 pg/ml.

### Immunoblotting

Rat primary microglia were left treated with RBE (50–300 μg/ml) for 24 h; then, the LPS (10 ng/mL) was added for different time points (depending on the studied protein). After the experiment, the cells were washed with cold PBS and lysed in the lysis buffer (42 mM Tris–HCl, 1.3 % sodium dodecyl sulfate, 6.5 % glycerin, 100 μM sodium orthovanadate, and 2 % phosphatase and protease inhibitors). Protein concentration of the samples was measured using the bicinchoninic acid (BCA) protein assay kit (Thermo Fisher Scientific, Bonn, Germany) according to the manufacturer’s instructions. For western blotting, 10–20 μg of total protein from each sample was subjected to sodium dodecyl sulfate-polyacrylamide gel electrophoresis (SDS-PAGE) under reducing conditions. Afterward, proteins were transferred onto polyvinylidene fluoride (PVDF) membranes (Millipore, Germany). After blocking with 5 % milk solution (BioRad, Munich, Germany) in Tris-buffered saline (TBS) containing 0.1 % Tween 20 (TBS-T), membranes were incubated with primary antibodies. Primary antibodies used were goat anti-COX-2 (1:500; Santa Cruz Biotechnology, Heidelberg, Germany), rabbit anti-mPGES-1 (1:6000; Agrisera, Vännas, Sweden), anti-inhibitor of kB (IkB)-α (1:500; Santa Cruz Biotechnology), anti-p44/42 (1:1000; Cell Signaling Technology, Frankfurt, Germany), anti-p38 (1:1000; Cell Signaling Technology), anti-JNK (1:1000; Cell Signaling Technology), anti-phospho p44/42 (1:1000; Cell Signaling Technology), anti-phospho-p38 (1:1000; Cell Signaling Technology), anti-phospho-JNK (1:1000; Cell Signaling Technology), and rabbit anti-actin (1:5000; Sigma Aldrich). Primary antibodies were diluted in TBS-T and 5 % BSA. Membranes were incubated with the primary antibody overnight at 4 °C followed by incubation in secondary antibodies. After extensive washing (three times for 10 min each in TBS containing 0.1 % Tween 20), proteins were detected with either horseradish peroxidase (HRP)-coupled anti-goat IgG (Santa Cruz Biotechnology) or anti-rabbit IgG (R&D systems, Wiesbaden-Nordenstadt, Germany) using enhanced chemiluminescence (ECL) reagents (GE Healthcare, Freiburg, Germany). Densitometric analysis was performed using ImageJ software (NIH, USA), and a β-actin control was used to confirm equal sample loading and normalization of the data.

### Real-time quantitative PCR

Quantitative real-time PCR (qPCR) was performed to determine the transcriptional regulation of COX-2, mPGES-1, TNF-α, IL-1β, IL-6, and IL-10 by RBE in activated microglia. RNA preparation was done by using RNAspin mini RNA isolation kit (GE Healthcare, Freiburg, Germany) and for cDNA synthesis, 1 μg of total RNA was reverse transcribed using M-MLV reverse transcriptase and random hexamers (Biomers, Ulm, Germany). The synthesized cDNA was the template for the real-time PCR amplification that was carried out by the CFX96 real-time PCR detection system (Bio-Rad Laboratories, Inc.) using iQ^TM^ SYBR^TM^ Green supermix (Bio-Rad Laboratories GmbH, Munich, Germany). Specific primers were designed by using Universal Probe Library Assay Design Center (Roche) and were obtained from Biomers (Ulm, Germany). Reaction conditions were 3 min at 95 °C, followed by 40 cycles of 15 s at 95 °C, 30 s at 50 °C, and 45 s at 72 °C, and every cycle was followed by plate reading. After that, 1 min at 95 °C, 1 min at 55 °C, followed by melt curve conditions of 65 °C, 95 °C with increment of 0.5C for 5 s, followed by final plate reading. Glyceraldehyde 3-phosphate dehydrogenase (GAPDH) served as an internal control for sample normalization, and the comparative cycle threshold Ct method was used for data quantification as described previously [36]. The following primer sequences were used in the present study. COX-2: Fwd 5′-GGCTTACAAGACGCCACATCACCT-3′; Rev 5′-TGGTTTAGGCGGCCGGGGAT-3′; mPGES-1: Fwd 5′-AGGCCAAGTCAGGCTGCGGA-3′; Rev 5′-GTCGTTGCGGTGGGCTCTGAG-3′; TNF-α: Fwd 5′-CCCACGTCGTAGCAAACCACCA-3′; Rev 5′-CCATTGGCCAGGAGGGCGTTG-3′; IL-1β: Fwd 5′-TGTGATGAAAGACGGCACAC-3′; Rev 5′-CTTCTTCTTTGGGTATTGTTTGG-3′; IL-6: Fwd 5′-CCTGGAGTTTGTGAAGAACAACT-3′; Rev 5′-GGAAGTTGGGGTAGGAAGGA-3′; IL-10: Fwd 5′-AGTGGAGCAGGTGAAGAATGA-3′; Rev 5′-TCATGGCCTTGTAGACACCTT-3′; GAPDH: Fwd 5′-TGGGAAGCTGGTCATCAAC-3′; Rev 5′-GCATCACCCCATTTGATGTT-3′.

### Flow cytometry

Microglial cells were pre-incubated with RBE (50–300 μg/ml) for 24 h; thereafter, LPS (10 ng/ml) was added for the next 24 h. Flow cytometry-based cell cycle analysis was performed as previously described with few modifications [37]. After the end of the incubation period, the media was removed and cells were washed with warm PBS. Subsequently, cells were detached from the culture dishes using 0.05 % trypsin (incubated at 37 °C, 5 min) and were then suspended in medium containing 10 % FCS. Afterwards, the cells were centrifuged (1000 rpm, 5 min) and the pellet was suspended in PBS and fixed overnight with 70 % ethanol. The next day, permeabilization (0.05 % Triton X-100) and staining with propidium iodide (PI, 50 μg/ml; 0.1 mg/ml RNaseA in PBS) was performed by incubating the cells for 40 min at 37 °C. Thereafter, stained cells were centrifuged (at 1500 rpm for 5 min) and pellet was suspended in 500 μl of PBS for flow analysis. The DNA content was then quantified using a FACScaliburTM flow cytometer (BD Biosciences, San Jose, CA, USA) equipped with argon laser (488 nm). While running the cytometer, dot plots were displayed to identify the cells based on their physical parameters on the forward scatter light (FSC) vs. side scatter light (SSC). To evaluate cell cycle progression, samples were acquired with the FL-2 fluorescence channel set to a linear scale, which allows better characterization of the G0/G1 and G2/M DNA peaks. Pulse processing was used to exclude cell doublets and clumps from the analysis. This can be achieved by using pulse area vs. pulse width. For the analysis, a gate was set on the single cell population using pulse width vs. pulse area. Then, this gate was applied to the scatter plot to gate out obvious debris. Thereafter, gates were combined and applied to the PI histogram plot. To quantitate the percentage of cells in each cell cycle phase, markers were set within the analysis program.

### Statistical analysis

Statistical analyses were performed using Prism 5 software (GraphPad software Inc., San Diego, CA, USA). Values of all experiments were represented as mean ± SEM of at least three independent experiments. Raw values were converted to percentage and LPS (10 ng/ml) was considered as 100 %. Values were compared using one-way ANOVA with post hoc Student-Newman-Keuls test (multiple comparisons). The level of significance was set at *p < 0.05, **p < 0.01, and ***p < 0.001.

## Results

### RBE significantly reduced the LPS-induced PGE_2_ release without any detrimental effects on the viability of cells

**Fig. 1:**
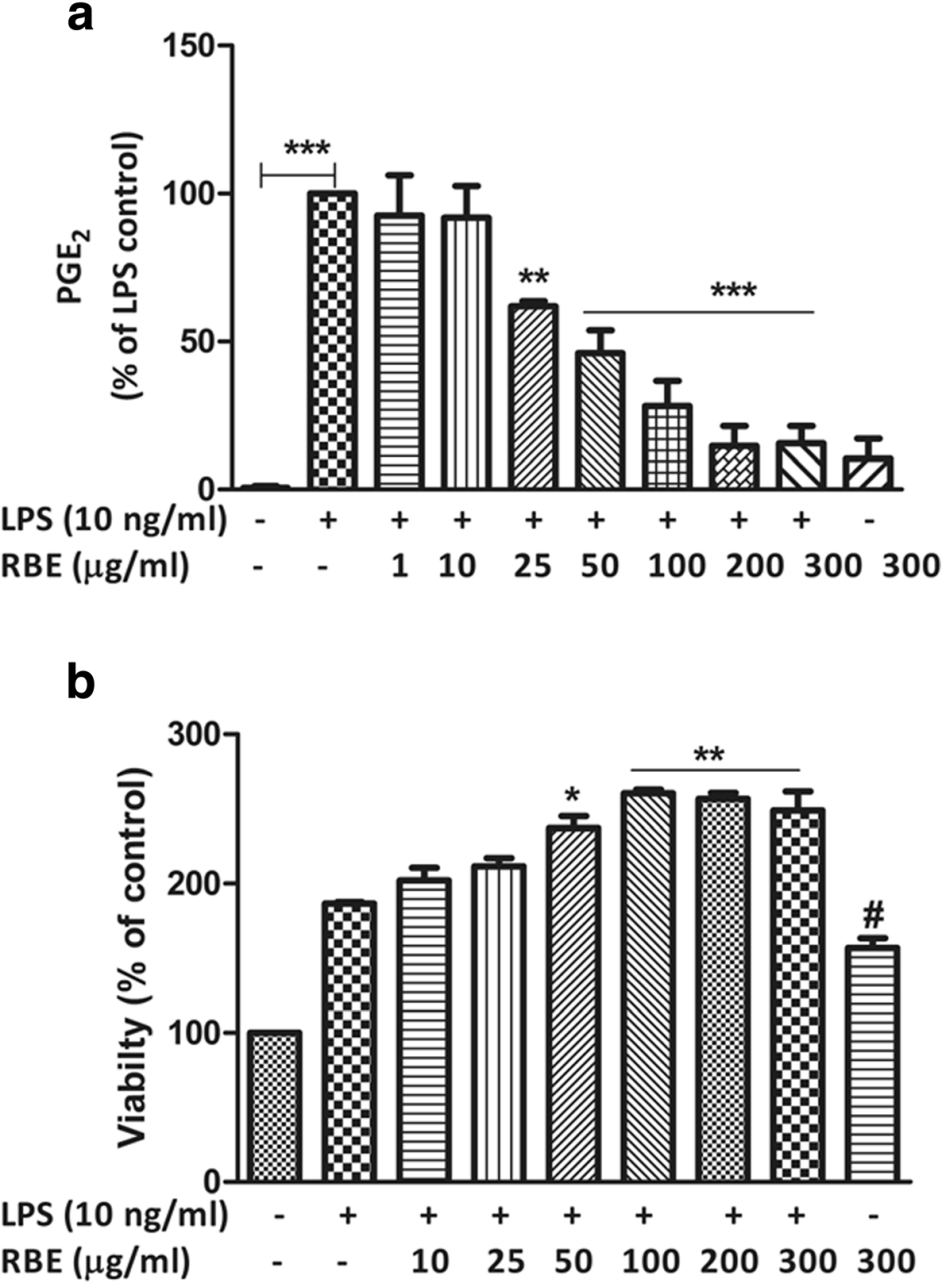
**a** Rice bran extract (RBE) inhibits the release of prostaglandin E_2_ (PGE_2_) in lipopolysaccharide (LPS)-activated microglia. **b** RBE did not exert any adverse effects on viability in LPS-stimulated microglia cells. Microglia were pre-treated with RBE (10–300 μg/ml) for 24 h; afterwards, cells were activated with or without LPS (10 ng/ml) for the next 24 h. At the end of incubation, cell supernatants were collected and release of PGE_2_ (**a**) was measured by enzyme immune assay (EIA). ATP assay was performed to assess the microglia cell viability after RBE (10–300 μg/ml) treatment in the absence or presence of LPS (10 ng/ml). For a detailed measurement protocol, see the “Methods” section. Statistical analyses were carried out by using one-way ANOVA with post hoc Student-Newman-Keuls test (multiple comparisons). Results are expressed as means ± SEM of at three independent experiments. ^*^ *p* < 0.05; ^**^ *p* < 0.01; ^***^ *p* < 0.001 compared with LPS (10 ng/ml)-activated cells; ^#^ *p* < 0.001 compared with control cells

To investigate whether RBE exerts anti-inflammatory effects, microglia cells were pre-incubated with RBE (10–300 μg/ml) for 24 h and then stimulated with or without LPS (10 ng/ml) for the next 24 h. As a result, we observed a marked increase in the production of PGE_2_ in the supernatants of microglia cells stimulated with LPS (6831 ± 799.0 pg/ml, *p* < 0.001) as compared to unstimulated cells (78.27 ± 24.27 pg/ml). Treatment with RBE for 24 h prior to stimulation with LPS resulted in a significant decrease in the release of PGE_2_ when compared with LPS (considered as 100 %). Significantly reduced levels of PGE_2_ were evident starting from the concentration of 25 μg/ml RBE (61.97 ± 1.70 %, *p* < 0.05), and a maximal decrease was observed at the concentration of 300 μg/ml (15.57 ± 5.90, *p* < 0.001) as shown in Fig. 1a. To rule out whether these effects were due to the decrease in the cell viability, ATP cell viability assay was performed. Obtained data showed that all the concentrations (ranging from 10 to 300 μg/ml) had no adverse effects on ATP levels and thus viability of the microglial cells. Control cells incubated with ethanol (used as solvent for RBE) at the end concentration of 0.15 % had no adverse effects on the viability. This conveys that reduction in the levels of PGE_2_ by RBE was not due to the cytotoxicity. More interestingly, the concentrations ranging from 50 to 300 μg/ml further elevated the levels of ATP in activated microglia as depicted in Fig. 1b. In addition, RBE (300 μg/ml) itself significantly increased the ATP levels under basal conditions (non-activated). Given the pro-proliferative function of vitamin E congeners [38], we suspected that increased levels of ATP by RBE might be due to its interference in the microglial cell cycle, increasing DNA content, hence affecting the viability of cells. Therefore, we performed cell cycle analysis by using DNA dye-propidium iodide (see Additional file 1: Figure S1(A-K)). As a result, we observed a tendency towards the enhancement of S and G2/M phases after 48-h incubation of RBE (300 μg/ml) in comparison with the control cells but, it did not reach to a statistical significant value (Additional file 1: Figure S1(K)). Since there was a further enhancement of ATP levels with RBE (threshold concentration of 50 μg/ml) in LPS-activated microglia (see Fig. 1b), we also performed assays with RBE (50–300 μg/ml) in activated microglia. Consequently, a trend (between RBE 100–300 μg/ml) towards an increase in percentage of cells in S and G2/M phases has seen as compare with LPS (10 ng/ml), indicating a minor enhancement of cell proliferation.

### RBE inhibits the free radical formation in LPS-induced rat microglia

**Fig. 2:**
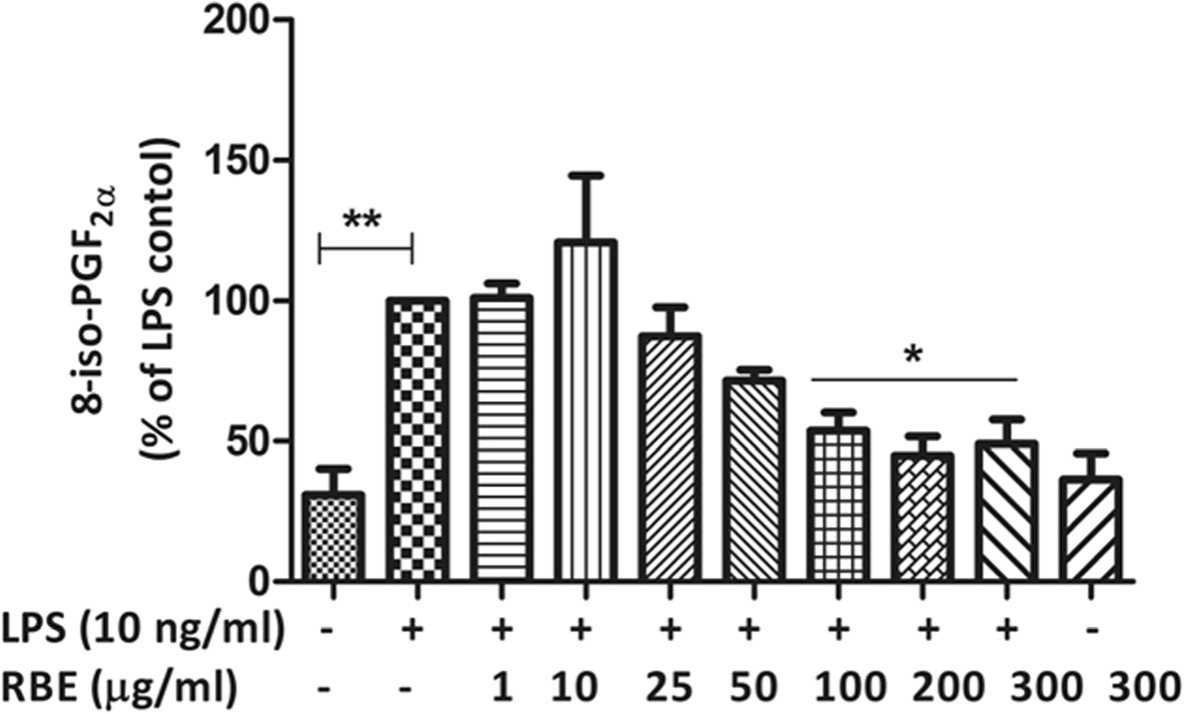
RBE inhibits the release of 8-isoprostane (8-iso-PGF_2α_) in LPS-activated microglia. Microglia cells were pre-treated with RBE (10–300 μg/ml) for 24 h; thereafter, cells were incubated with or without LPS (10 ng/ml) for the next 24 h. At the end of incubation, cell supernatants were collected and release of 8-iso-PGF_2α_ was measured by EIA. Statistical analysis was carried out by using one-way ANOVA with post hoc Student-Newman-Keuls test (multiple comparisons). Results are expressed as means ± SEM of three independent experiments. ^*^ *p* < 0.05 compared with LPS (10 ng/ml)-activated cells

Given the anti-oxidant properties of RBE [39], we also speculated that RBE might exert its anti-oxidative effects in activated microglia. To this end, we studied the effects of RBE on the formation of free radical formation in LPS-activated microglia. Measurement of 8-iso-PGF_2α_ release is taken as a sensitive marker to assess free radical formation [40], and we have previously shown that LPS significantly increases the levels of 8-iso-PGF_2α_ in primary microglia [41]. Indeed, here, we also observed that LPS (10 ng/ml) significantly enhanced the levels of 8-iso-PGF_2α_ (134.8 ± 12.08 pg/ml, *p* < 0.001) as compared to basal levels (44.15 ± 15.80 pg/ml). Pre-incubation of RBE for 24 h reduced the levels of released 8-iso-PGF_2α_ in LPS-activated microglia. This reduction in the levels was achieved ranging from the concentration of RBE 100 μg/ml (53.73 ± 6.57 %, *p* < 0.05), RBE 200 μg/ml (44.97 ± 6.76 %, *p* < 0.05), and 300 μg/ml (49.16 ± 8.54 %, *p* < 0.05). RBE did not show any effect in the formation of isoprostane under control conditions (Fig. 2).

### RBE supresses the PGE_2_ production by inhibiting the COX-2 and mPGES-1 expressions in LPS-activated microglia

**Fig. 3:**
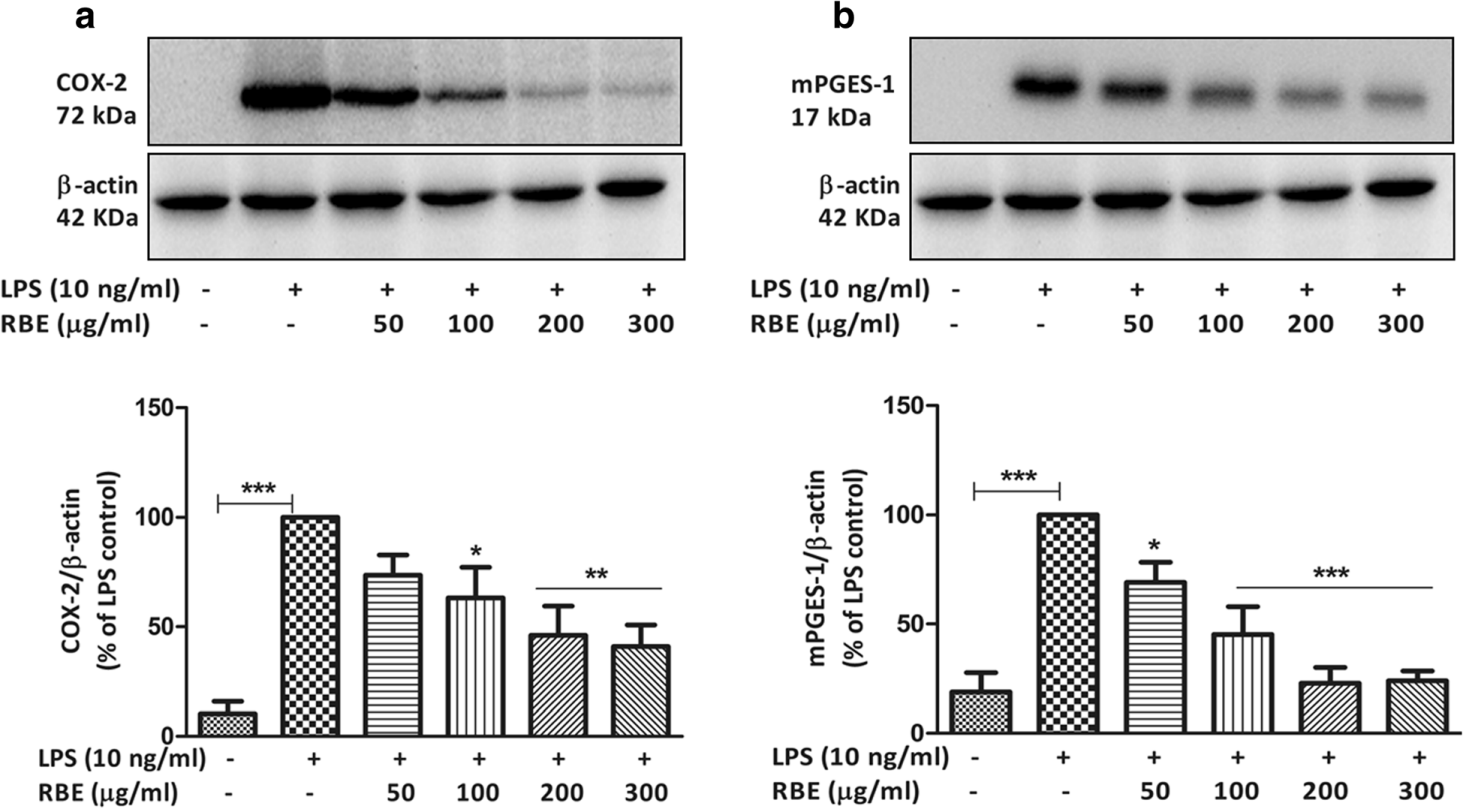
Effects of RBE on protein levels of COX-2 and mPGES-1 in LPS-activated microglia. Cells were pre-treated with RBE (50–300 μg/ml) for 24 h followed by incubation of cells with or without LPS (10 ng/ml) for the next 24 h. Whole cell lysates were subjected to western blot for COX-2 and mPGES-1. Representative blots for COX-2 and mPGES-1 are shown (**a**, **b**, *upper panel*) and densitometric analyses were performed (**a**, **b**, *lower panel*). To confirm equal sample loading, membranes were stripped and reprobed for β-actin and the data were used for normalization. Statistical analyses were carried out by using one-way ANOVA with post hoc Student-Newman-Keuls test (multiple comparisons). Results are expressed as means ± SEM of three to five independent experiments. ^*^ *p* < 0.05; ^**^ *p* < 0.01; ^***^ *p* < 0.001 compared with LPS (10 ng/ml)-activated cells

PGE_2_ is synthesized during microglia activation through the enzymatic action of COX-2 and mPGES-1. It is known that mPGES-1 is coupled with COX-2 in the biosynthesis of PGE_2_. Consequently, we determined whether the effect of RBE on PGE_2_ was mediated through an inhibition of the synthesis of these enzymes. As shown in Fig. 3a, RBE (50 μg/ml) did not cause a significant reduction in COX-2 protein expression in activated (LPS 10 ng/ml) microglia. However, significant reduction in COX-2 immunoreactivity was observed initially at 100 μg/ml and was strongly inhibited at 200 and 300 μg/ml. On the contrary, inhibition of mPGES-1 protein levels started already with the lowest concentration of 50 μg/ml, stronger effects were seen at 100 μg/ml, and maximal effects were achieved at 200 μg/ml as compared to 300 μg/ml as shown in Fig. 3b. However, RBE did not significantly alter the expression of COX-2 (only a marginal increase observed) and mPGES-1 under non-activated state of microglia (Additional file 2: Figure S2 and Additional file 3: Figure S3 (B)). These data suggest that reduction in the release of PGE_2_ by RBE is due to decreased expression of COX-2 and mPGES-1.

### Inhibition of p38 MAPK, ERK1/2, and JNK activation contributes to the anti-inflammatory effects of RBE

**Fig. 4:**
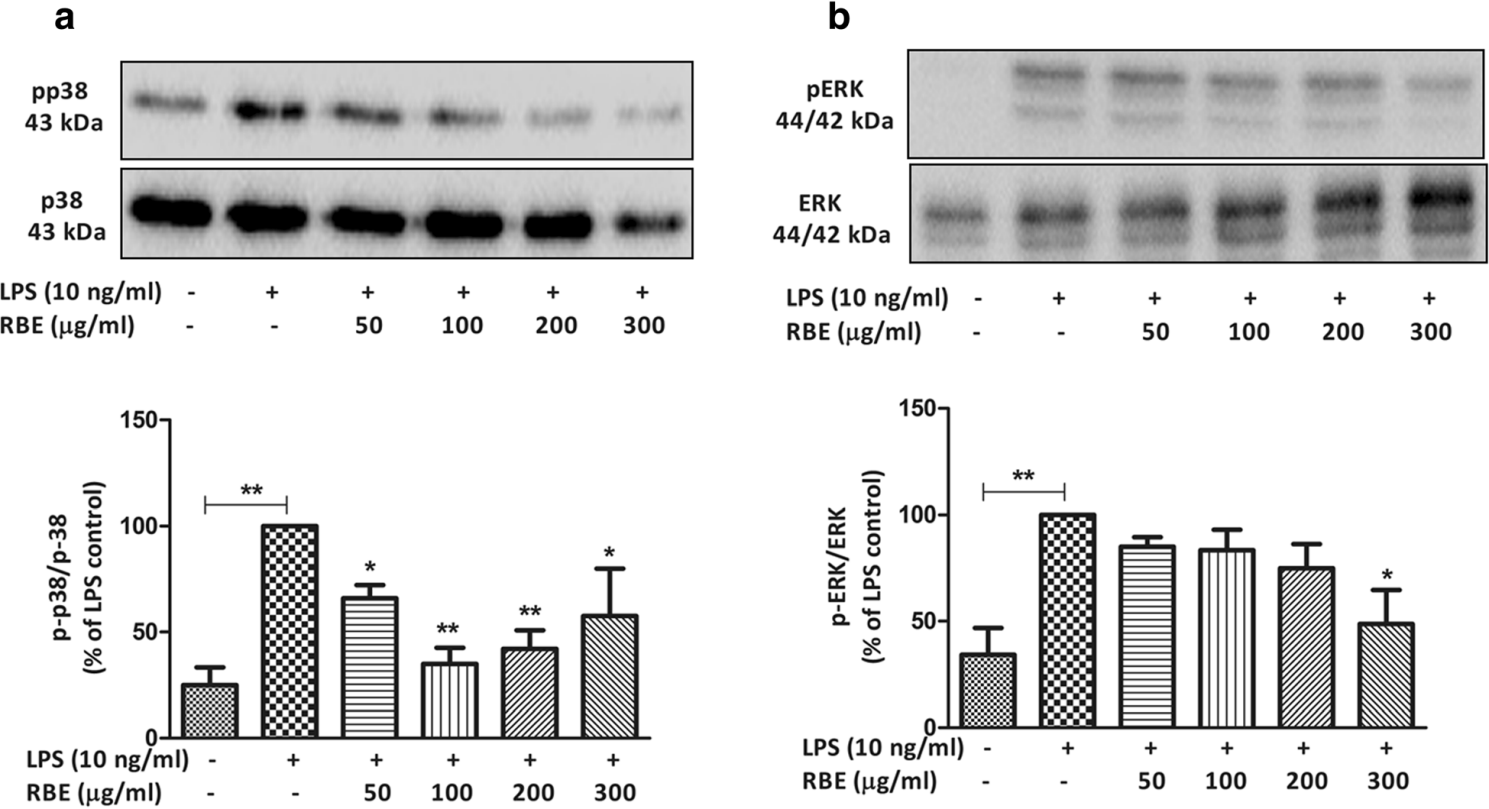
Effects of RBE on the activation of p38 MAPK and ERK 1/2 MAPK. Cells were pre-treated with RBE (50–300 μg/ml) for 24 h followed by stimulation with or without LPS (10 ng/ml) for 30 min. Whole cell lysates were subjected to western blots analyses. Representative blots (*upper panel*) and densitometry analyses (*lower panel*) are shown **a** p38 MAPK and **b** ERK 1/2. Statistical analyses were carried out by using one-way ANOVA with post hoc Student-Newman-Keuls test (multiple comparisons). Results are expressed as means ± SEM of three to four independent experiments. ^*^ *p* < 0.05; ^**^ *p* < 0.01; ^***^ *p* < 0.001 compared with LPS (10 ng/ml)-activated cells

The role of MAPKs in neuroinflammation and Alzheimer’s disease is well evident [21, 42, 43]. The major MAPK pathway subfamilies involved in the regulation of COX-2/mPGES-1 and cytokine synthesis in LPS-activated microglia are signaling proteins such as extracellular signal-regulated kinases (ERK1/2) or (p44/42 MAPK), JNK, and p38 MAPK. Therefore, we studied the possible effects of RBE on the phosphorylation of these kinases in primary rat microglia. As a result, pre-incubation of RBE for 24 h has differential effects on the phosphorylation of p38, ERK1/2, and JNK in LPS (10 ng/ml)-activated microglia. Interestingly, reduction in the phosphorylation of p38 with RBE was achieved as low as with 50 μg/ml (~35 %, *p* < 0.05), which was further reduced (~60 %, *p* < 0.01) with higher concentrations (RBE 100 and 200 μg/ml). The phosphorylated levels remained significantly low with the highest concentration of RBE (300 μg/ml), (~40 %, *p* < 0.05) as shown in Fig. 4a

**Fig. 5:**
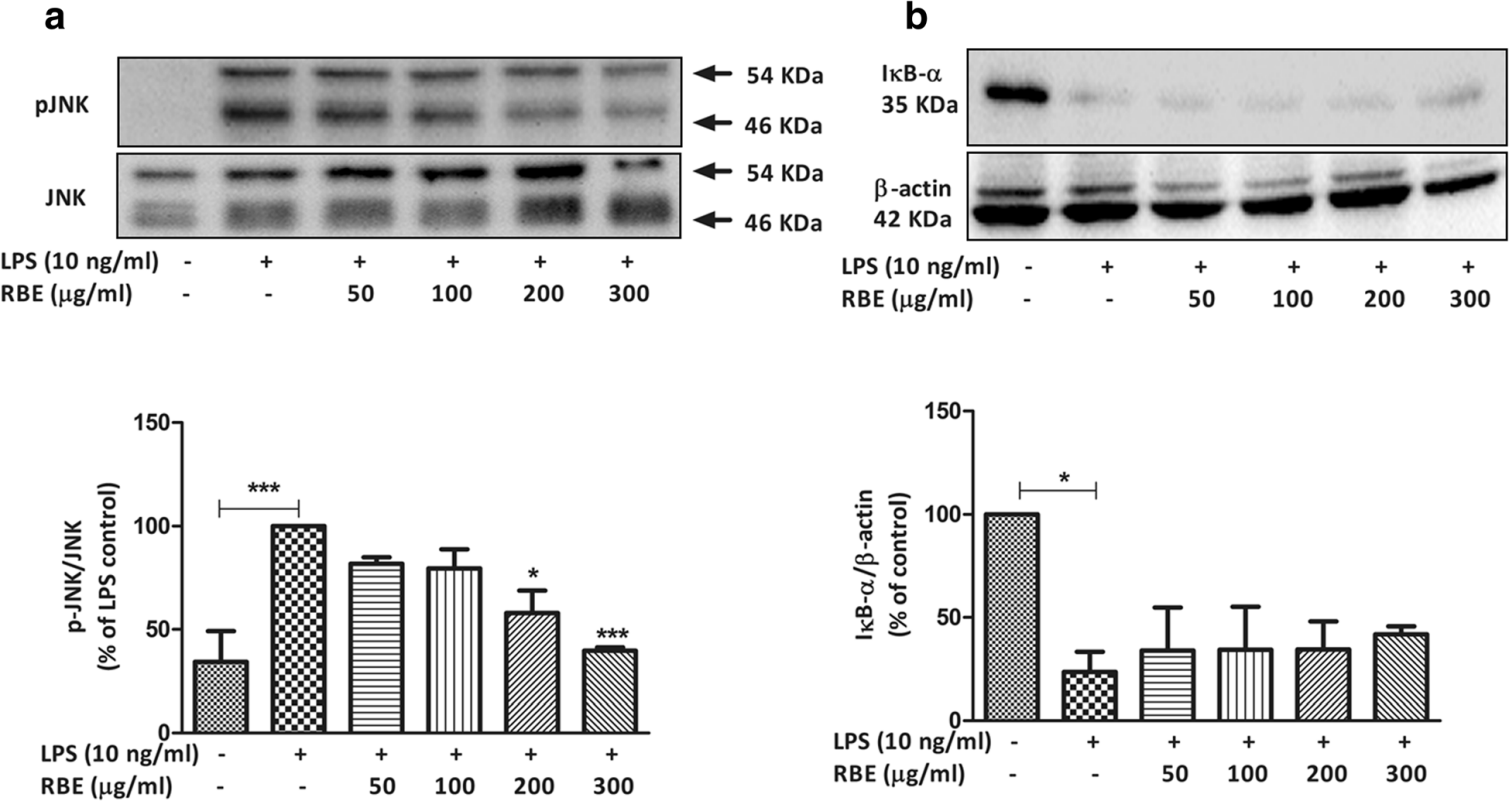
Effects of RBE on the activation of JNK and degradation of IkB-α induced by LPS. Cells were pre-treated with RBE (50–300 μg/ml) for 24 h followed by stimulation with or without LPS (10 ng/ml) for 30 min (for phospho-JNK) and 15 min (for IkB-α). Whole cell lysates were subjected to western blots analyses. Representative blots (*upper panel*) and densitometry analyses (*lower panel*) are shown **a** pJNK and **b** IkB-α. Statistical analyses were carried out by using one-way ANOVA with post hoc Student-Newman-Keuls test (multiple comparisons). Results are expressed as means ± SE of three three to four independent experiments. ^*^ *p* < 0.05; ^**^ *p* < 0.01; ^***^ *p* < 0.001 compared with LPS (10 ng/ml)-activated cells or control cells (for IkB-α graph)

As shown in Fig. 4a, significant reduction of pERK (~50 %, *p* < 0.05) was only achieved with the highest concentration of RBE (300 μg/ml). However, only a tendency towards decrease was observed with lower concentrations but did not reach a significant statistical value (Fig. 4b). Furthermore, RBE (200 μg/ml) exerted inhibition of ~40 % (*p* < 0.05) and RBE (300 μg/ml) ~60 % (*p* < 0.001) of the activation of JNK in microglia (Fig. 5a). Furthermore, we also studied if RBE itself has any significant influence on the phosphorylation of the abovementioned kinases. Similarly, RBE did not alter the phosphorylation of p38MAPK, ERK (only marginal increase but not significant), and JNK under non-activated state of microglia (Additional file 4: Figure S4 (A-C)).

### Anti-inflammatory effects of RBE were independent of IkB-α degradation

One of the first responses of the microglia to LPS stimulation is the phosphorylation and degradation of IkB. In resting cells, NF-kB is sequestered in the cytoplasm by the inhibitory IkB protein. When activated by a stimulus, such as LPS, IkB is phosphorylated by IKK. Phosphorylated IkB then undergoes ubiquitination and degradation [44] and allows the NF-kB to translocate to the nucleus and thereby facilitates the transcription of various pro-inflammatory genes. The decay of IkB-α is used as an indicator of NF-kB activation [35, 45]. Therefore, we also studied the effects of RBE on the degradation of IkB-α. Stimulation of LPS (10 ng/ml) for 15 min led to the degradation of IkB-α while pre-incubation of RBE for 24 h failed to reverse this effect (Fig. 5b), suggesting that the regulatory effects of RBE on microglia activation are confined to the MAPK pathway.

### Effects of RBE on the LPS-mediated synthesis of pro-inflammatory and anti-inflammatory cytokines

**Fig. 6:**
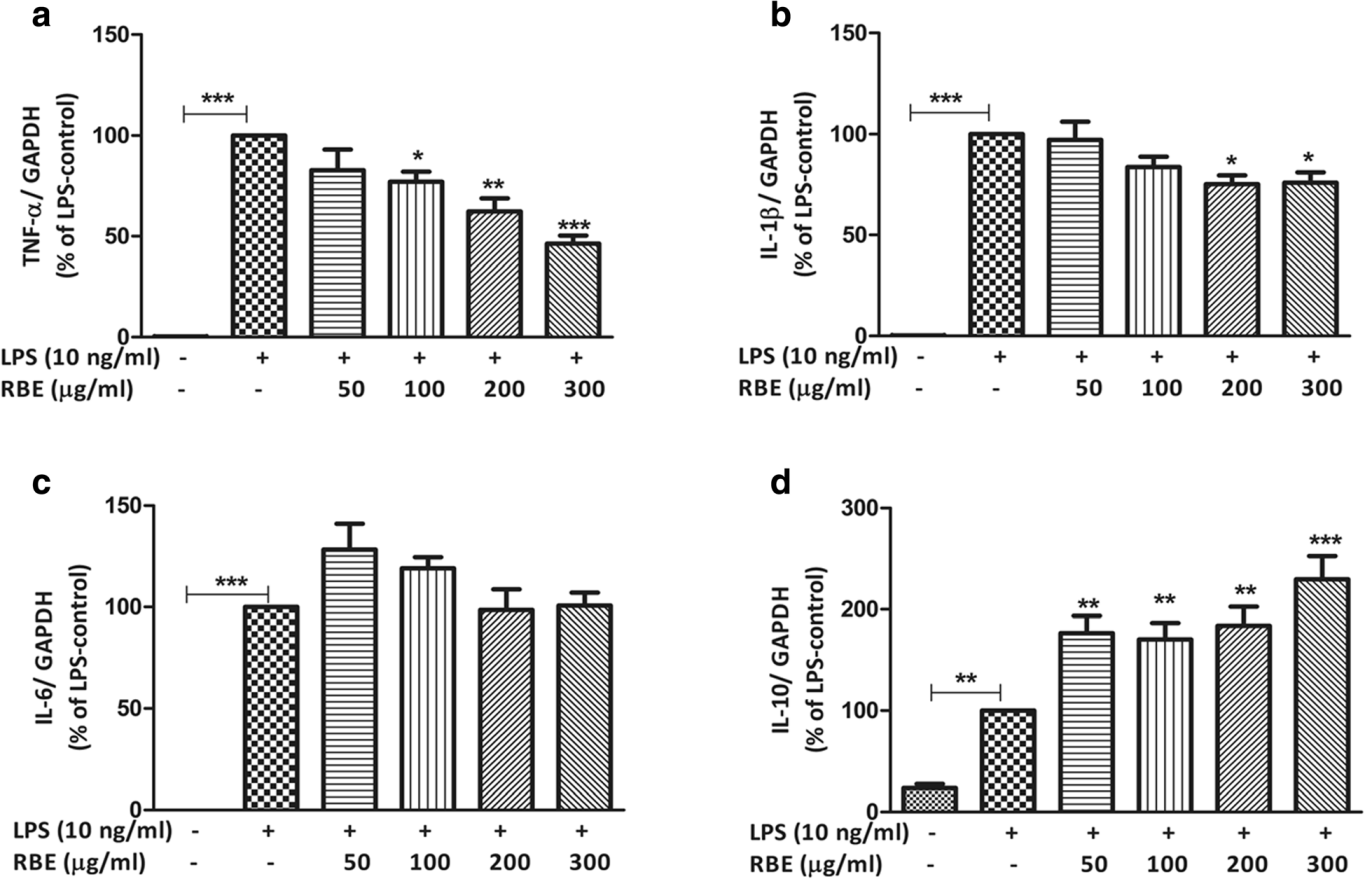
Effects of RBE on the gene expression of TNF-α, IL-1β, IL-6, and IL-10 in LPS-activated microglia. Cells were pre-treated with RBE (50–300 μg/ml) for 24 h followed by stimulation with or without LPS (10 ng/ml) for 4 h. Afterwards, gene expression of **a** TNF-α, **b** IL-1β, **c** IL-6, and **d** IL-10 was analyzed by real-time quantitative PCR. GAPDH was used as an internal control for normalization and data were quantified by using comparative cycle threshold Ct method. Data are presented as percentage control of LPS. Results are expressed as means ± SEM of three to five independent experiments. Statistical analyses were carried out by using one-way ANOVA with post hoc Student-Newman-Keuls test (multiple comparisons). ^*^ *p* < 0.05; ^**^ *p* < 0.01; ^***^ *p* < 0.001 compared with LPS (10 ng/ml)-activated cells

**Fig. 7:**
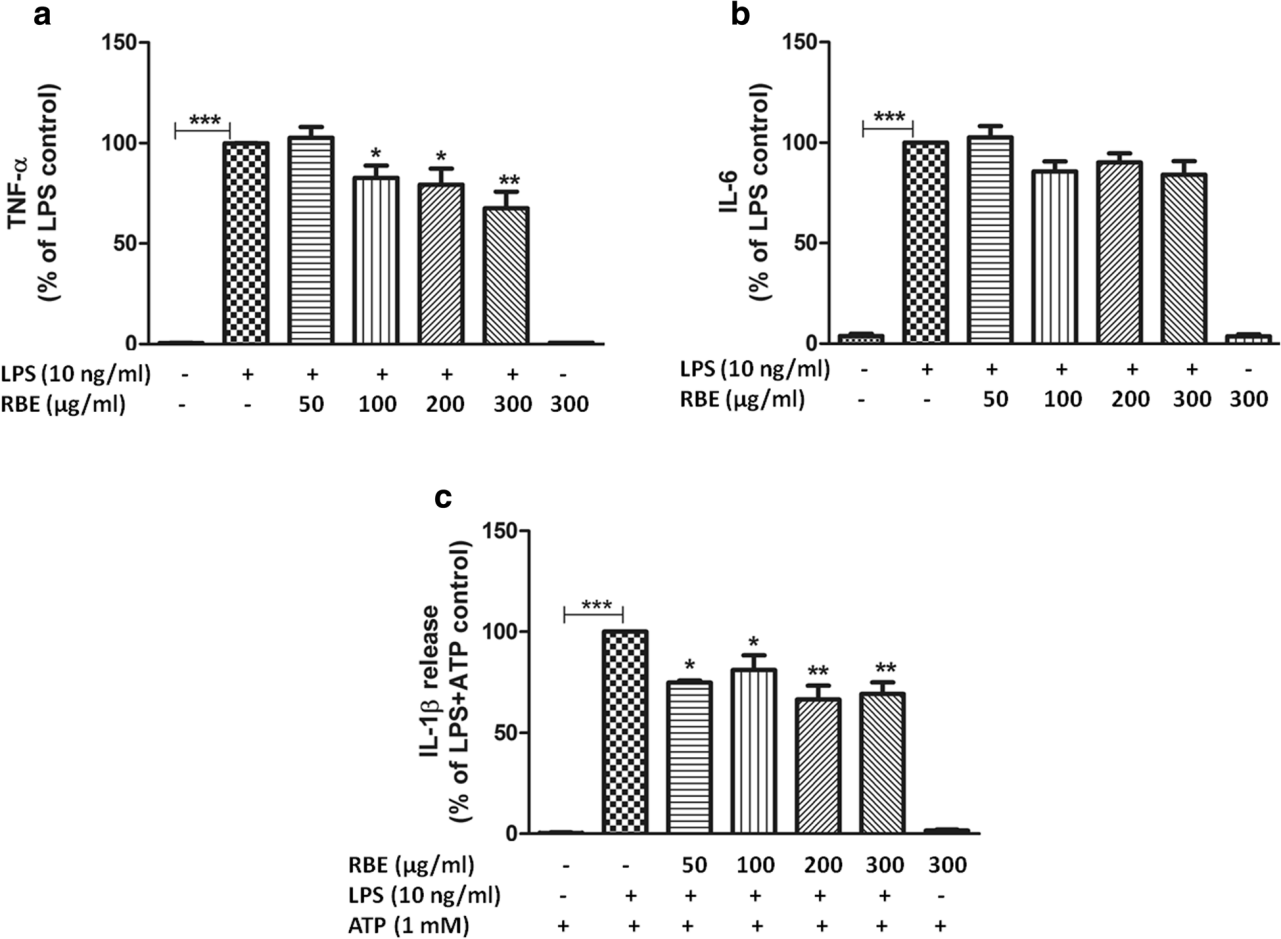
Effects of RBE on the release of TNF-α, IL-1β, and IL-6 in LPS-activated microglia. Cells were pre-treated with RBE (50–300 μg/ml) followed by stimulation with or without LPS (10 ng/ml) for 24 h. Afterwards, release of **a** TNF-α, **b** IL-1β, and **c** IL-6 was analyzed by ELISA. Data are presented as percentage control of LPS. Results are expressed as means ± SEM of three to four independent experiments. Statistical analyses were carried out by using one-way ANOVA with post hoc Student-Newman-Keuls test (multiple comparisons). ^*^ *p* < 0.05; ^**^ *p* < 0.01; ^***^ *p* < 0.001 compared with LPS (10 ng/ml)-activated cells

We furthermore tested whether the effects of RBE were confined to the reduction of PGE_2_ and isoprostane release or if they also affect other inflammatory molecules. To this end, we studied the effects of RBE on the gene expression as well as release of pro-inflammatory cytokines (TNF-α, IL-1β, and IL-6). These cytokines are known to be important mediators of microglia inflammation [35]. Stimulation of microglia cells with LPS (10 ng/ml) for 4 h led to a significant increase in normalized expression of TNF-α (LPS, 1.75 ± 0.21 vs. control, 0.005 ± 0.0005, *p* < 0.001), IL-1β (LPS, 1.31 ± 0.067 vs. control, 0.003 ± 0.0007, *p* < 0.001), and IL-6 (LPS 0.91 ± 0.09 vs. control 0.0 ± 0.0, *p* < 0.001) as shown in Fig. 6a–c. Pre-treatment of RBE for 24 h significantly reduced the expression of TNF-α and IL-1β but failed to impart any effect on the expression of IL-6 (Fig. 6c). Mild reduction in the expression of TNF-α started at 100 μg/ml of RBE (77.07 ± 4.96 %, *p* < 0.05), moderate at 200 μg/ml (62.27 ± 6.59 %, *p* < 0.01) and achieved maximal effects at 300 μg/ml (46.31 ± 3.96 %, *p* < 0.001) as depicted in Fig. 6a. Pre-treatment of RBE (50 and 100 μg/ml) did not show any significant modulatory effects on the expression of IL-1β (Fig. 6b). However, with the higher concentrations of RBE (200 and 300 μg/ml), a significant decrease of ~25 % (*p* < 0.05) was observed as compared with LPS 10 ng/ml (taken as 100 %). Furthermore, in case of release of cytokines, 24-h stimulation of microglia with LPS (10 ng/ml) leads to a significant upregulation of TNF-α (control, 7.71 ± 2.57 pg/ml vs. LPS, 1401 ± 130 pg/ml), IL-6 (control, 52.73 ± 2.64 pg/ml vs. LPS, 1086 ± 88.40 pg/ml), and IL-1β (control, 1.35 ± 1.35 pg/ml vs. LPS + ATP, 316.8 ± 87.01 pg/ml). Similarly, cells with the prior treatment of RBE had less levels of TNF-α and IL-1β whereas levels of IL-6 did not alter significantly as shown in Fig. 7a–c. Of note, in case of IL-1β release, addition of ATP (30 min prior to collection of supernatants from culture wells) was important since release of IL-1β is dependent on the ICE/caspase 1. ATP via the P2X7 receptor acts as extracellular trigger for ICE/caspase 1 activation. Therefore, we did not see any significant changes in the extracellular accumulation of IL-1β with LPS (10 ng/ml, for 24 h) in the absence of ATP (data not shown in the graph). All of these above data suggest specific effects of RBE (at various threshold concentrations) on distinct inflammatory mediators in microglia. In order to understand if RBE has any modulatory effects on the polarity of microglia (M1, pro-inflammatory vs. M2, anti-inflammatory), gene expression of the well-accepted M2 cytokine (IL-10) was studied using real-time PCR. In contrast to pro-inflammatory cytokines, treatment of RBE led to further augmentation of IL-10 gene expression starting from RBE 50 μg/ml (176.7 ± 17.07 %, *p* < 0.01), at RBE 100 μg/ml (170.3 ± 16.36 %, *p* < 0.01), at RBE 200 μg/ml (183.9 ± 18.79 %, *p* < 0.01), and reached a maximum at RBE 300 μg/ml (229.5 ± 23.22 %, *p* < 0.001) as shown in Fig. 6d. These data indicate that RBE might be involved in polarizing microglia by influencing the expression of pro- and anti-inflammatory cytokines. Additionally, to seek the experimental evidences if these beneficial effects of RBE in microglia can be mimicked by α-tocopherol (one of the active components of RBE), various assays have been performed. Briefly, experiments were performed with α-tocopherol (10–100 μM) in a similar manner as of RBE to see if α-tocopherol can mimic all or some of the data we got with RBE treatment. Interestingly, we did not observe any significant changes in the levels of PGE_2_, TNF-α, IL-1β, and IL-6 (see Additional file 5: Figure S5 (A-D)). These data indicates that effects mediated by RBE in microglia cannot be explained by the α-tocopherol alone.

**Fig. 8:**
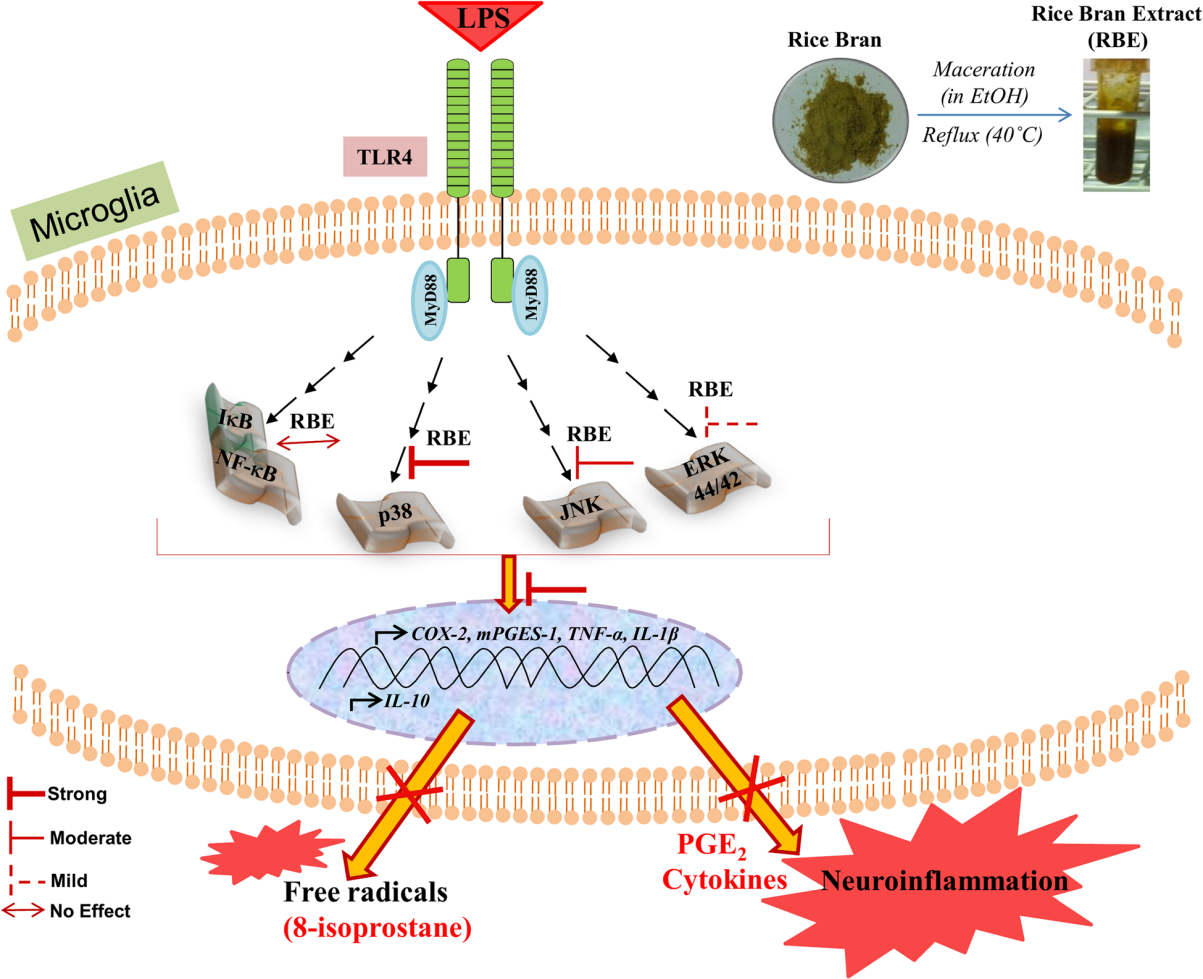
Schematic showing the effects of RBE on LPS-activated microglia. Stimulation of Toll-like receptor (TLR)-4 by LPS resulted in a phosphorylation and thus activation of mitogen-activated protein kinases (MAPK) signal cascade as well as NF-kB pathway. RBE exerted its beneficial effects by interfering in the activation of key kinases in MAPK signaling cascade but failed to affect NF-kB pathway. These effects of RBE were corroborated by reduction in the levels of COX-2/mPGES-1 and hence PGE_2_ production. RBE also imparted its anti-inflammatory effects by reducing the expression of pro-inflammatory cytokines (M1 markers; IL-1β and TNF-α) and by counterbalancing the levels of anti-inflammatory cytokine (M2 marker; IL-10) in microglia. Moreover, these beneficial effects of RBE are possibly due to, at least in part, by reduction in free radical formation (8-isoprostane) in microglia

## Discussion

In this study, we demonstrate that RBE inhibits microglia activation by suppressing the production of PGE_2_ and 8-iso-PGF_2α_—a marker for free radical production. RBE exhibited further beneficial effects by reducing pro-inflammatory cytokines (TNF-α and IL-1β). In addition, pre-stimulation with RBE for 24 h enhanced the expression levels of anti-inflammatory (IL-10) cytokine in LPS-stimulated rat microglia. Furthermore, we were also able to dissect the underlying signaling mechanism/s by which RBE imparted its beneficial effects, schematically represented in Fig. 8. To the best of our knowledge, this is the very first evidence for the anti-inflammatory effects of RBE in the immune cells of CNS. Immunomodulatory effects of rice bran (black and brown) extract in the peripheral nervous system (PNS) of CD-1 mice have previously been explored [46]. This study showed the anti-inflammatory activities of black rice bran (10 mg/kg body weight) in a topical application of TPA (12-*O*-tetradecanolylphorbol-13-acetate)-induced skin edema. Anti-inflammatory actions exerted by black rice bran in this study were due to the suppression of TNF-α, IL-6, and IL-1β leaving PGE_2_ production unchanged, whereas brown rice bran extract had no effect on studied molecules. This study is in contrast to ours, at least in part, where we showed significant downregulation of PGE_2_ without affecting the IL-6 production. This inconsistency in studies might be explained due to the different study models (CNS vs. PNS; in vitro vs. in vivo). Another explanation might lie in the different composition and stability of RBE due to the distinct extraction methods. We observed that RBE did not reduce the survival of microglial cells either alone or in combination with LPS. More interestingly, RBE further elevated the levels of ATP under basal as well as in stimulated conditions. These findings are in agreement with the previous findings where the authors showed the beneficial effects of RBE on mitochondrial dysfunction. These activities of RBE were attributed to restoring mitochondrial endogenous respiration and ATP contents [31, 32]. One can speculate that restoration of mitochondrial function by RBE was owing to its anti-inflammatory and anti-oxidant features. There are few evidences showing that mitochondrial function may require a multifaceted approach that includes drugs and plant-derived phenolic compounds with anti-oxidant and anti-inflammatory activities [47]. It is not precisely clear if such correlation exists in our model, and we believe that this is intriguing and worth exploring in our future studies. Another reason for the increase in ATP levels might be due to an enhancement in the proliferation of microglia after RBE treatment. To find this out, we performed cell cycle analysis. As a result, we did observe a tendency towards an enhancement of microglial proliferation with RBE but it did not reach to a significant statistical value. Thus, we believe that this enhancement in proliferation might have contributed towards enhanced levels of ATP as shown in viability assay but we cannot rule out other factors, which warrant further assessment. Moreover, to the best of our knowledge, there is no such study investigating the proliferation after RBE treatment in the brain (in vivo) or in glial cell cultures. Though in contrast, there are previous studies showing the anti-proliferative actions of rice bran or RBE in cancerous cell lines. For example, water-soluble enzymatic extract from rice bran (EERB) imparted its anti-proliferative effects in human T cell acute lymphoblastic leukemia (MOLT-4) [48]. Another study by a Japanese group showed the immunomodulatory and anti-proliferative effects of various rice bran extracts (red, black, brown) in culture immunocompetent cells [49]. All of these studies have various experimental differences in comparison with our study. These studies were done with much higher concentrations of RBE (e.g., 300 μg/ml vs. 10 mg/ml); another differences were primary cell cultures vs. cell lines and differences in the variety of rice bran and preparation of extract. If we look at the effects of vitamin E congeners (tocopherols and tocotrienols) on the proliferation, there are ample evidences proving the pro-proliferative effects. For example, Ren et al. showed that tocotrienols induce lymphocyte proliferation, and the study by Flanary and Streit showed that α-tocopherol induces proliferation in cultured rat microglia [38, 50]. Thus, in our study, this enhancement of proliferation of microglia might have come through tocopherol or tocotrienols or via synergistic effects of both.

Next, we examined the effects of RBE on the formation of 8-iso-PGF_2α_—an index of oxidative stress-mediated production of lipid hydroperoxides. In postmortem brains of AD patients, levels of F2-isoprostane have been shown to be elevated [51, 52]. Earlier, we have demonstrated the ability of natural products to inhibit the 8-iso-PGF_2α_ in activated microglia [41, 53]. Similarly, our current findings with RBE show that the extract affects the formation of free radicals in microglia, confirming the anti-oxidant properties of this extract.

The COX-2/mPGES-1 enzyme pathway and subsequent generation of prostaglandins play a significant role in microglia activation. mPGES-1 is the terminal enzyme for the biosynthesis of PGE_2_ during inflammation and is normally functionally coupled with COX-2 with few exceptions [18]. This enzyme is markedly induced by pro-inflammatory stimuli and is downregulated by anti-inflammatory glucocorticoids. In order to gain a better understanding of the mechanism of action of RBE, we investigated its effect on COX-2 and mPGES-1 protein levels in LPS-treated microglia. Our data show that RBE inhibited COX-2 and mPGES-1 after 24 h of LPS stimulation, suggesting that RBE acts to reduce PGE_2_ production by interfering with both COX-2 and mPGES-1 protein synthesis in LPS-activated microglia. Previously, the stabilized RBE has shown to inhibit the activity of COX-2 enzyme in a cell-free system [54]. In this study, an effect of RBE on the mPGES-1 was not studied, suggesting the novelty of our work.

MAPK are involved in the regulation of inflammatory events. For instance, activation of ERK and p38 MAPK are implicated in the regulation of COX-2 expression in activated microglia [15, 55]. Moreover, our group has previously shown the involvement of ERK and p38 MAPK in the induction of mPGES-1 in rat microglia [18]. In this study, a common involvement of JNK has also been reported in the regulation of both COX-2 and mPGES-1 expression. In our present study, RBE exerted strong suppressive effects on the phosphorylation of p38 and JNK following LPS induction. However, in case of pERK, only modest inhibitory effects were seen with the highest concentration of RBE (300 μg/ml). Differential regulation of these proteins at various threshold concentrations of RBE indicates specificity of its ingredients for certain proteins. For instance, at the same time, one of the ingredients has inhibitory effects on certain protein while enhancing effect on others.

NF-kB activation has been linked to neurotoxicity through production of pro-inflammatory molecules in glial cells, suggesting its role in neurodegenerative conditions [56]. We therefore determined if RBE exhibited its anti-inflammatory effects via the suppression of NF-kB activation. The inflammatory responses to NF-kB are negatively regulated by its inhibitory subunit IkB-α [57]. In the current study, we found that LPS treatment reduced IkB-α levels in microglia as compared to untreated cells while pre-incubation of RBE for 24 h did not affect IkB-α degradation. However, the fact that RBE did not affect LPS-induced IkB-α degradation suggests that reduced expression of COX-2 and mPGES-1 by RBE is independent of NF-kB activation. Most recently, the bioavailability of RBE components in the brains of Guinea pigs and aged NMRI mice were examined [31, 58]. Apart from this there are—to the best of our knowledge—no more publications about bioavailability of RBE ingredients after administration of RBE. Briefly, they found that α-tocopherol was the most abundant vitamin E derivative found in Guinea pig brains, followed by α-tocotrienol and γ-tocotrienol. Another study was performed in NMRI—one of the well-established models of senescence. Similarly, only α-tocopherol could be detected in brain homogenates of young and aged mice and concentrations of all other tocopherol and tocotrienol derivatives were below the limit of detection. Therefore, to seek the experimental evidences if these effects imparted by RBE can be mimicked by α-tocopherol, we performed various assays with α-tocopherol. Interestingly, we did not observe any significant changes in the levels of PGE_2_, TNF-α, IL-1, and IL-6 (see Additional file 5: Figure S5 (A-D)). These data indicates that effects mediated by RBE in microglia cannot be explained by the α-tocopherol alone. RBE is a complex blend of known as well as still unknown compounds. Thus, we think that components other than α-tocopherols might have exerted their effects in regulating microglial activation. Previously, it was shown that only vitamin E from natural sources like RBE (mixture of eight vitamin E congeners) was able to protect brain against neurodegeneration [23, 59]. Accordingly, we can hypothesize that α-tocopherol is not the only active component present in RBE; others, most importantly tocotrienols, may have also contributed towards the downregulation of microglial activation. Also, one cannot rule out the effects of polyunsaturated fatty acids (PUFA) such as alpha-linolenic acid, which is also present in RBE. Studies on diets and drugs targeting PUFA have been emerging as disease-modifying agents [60]. We believe that our present study provides the fundamental knowledge (for us and others) of effects of RBE in regulating microglial function. Thus, a more elaborated characterization of RBE contents and its accumulation in the brain is required to identify the function of particular active compound.

## Conclusions

Altogether, our data provided the evidence that in activated microglia, RBE exerts beneficial effects by targeting COX-2/mPGES-1 in PGE_2_ production as well as by modulating cytokine levels. These beneficial effects are possibly due to, at least in part, anti-oxidant features of the extract. The underlying mechanism of action by which RBE exerted its anti-inflammatory effects was mainly dependent on MAPKs pathway without affecting NF-kB signaling. Thus, RBE represents the potential to regulate microglia activation (in vitro) and must be further investigated in animal models of neuroinflammation.

## Abbreviations

8-iso-PGF_2α_, 8-iso-prostaglandin F_2α_; COX-2, cyclooxygenase-2; ERK 1/2, extracellular signal-regulated kinase; IkB, inhibitor of kB; IL, interleukin; JNK, c-Jun N-terminal kinase; LPS, lipopolysaccharide; MAPK, mitogen-activated protein kinase; mPGES-1, microsomal prostaglandin E synthase-1; PGE_2_, prostaglandin E_2_; RBE, rice bran extract; TNF, tumor necrosis factor

## Additional files

Additional file 1: Figure S1. (A-K). Possible effects of rice bran extract (RBE) on the proliferation of microglia. Microglia were treated with either RBE (300 μg/ml) alone or RBE (50–300 μg/ml) in combination with LPS (10 ng/ml) for a total of 48 h. Thereafter, samples were stained with propidium iodide and processed for proliferation assay (for detailed protocol, see the “Methods” section) by using flow cytometer. Samples were acquired with the FL-2 fluorescence channel set to a linear scale, in order to amplify the diploid DNA peak. Graph A) represents the dot plot of cells acquired on the basis of side scattered light (SSC) and forward scattered light (FSC) and B) represents the pulse processing by using pulse area vs. pulse width. Graphs C-I) are representative histograms after each treatment. Markers represent the percentage of cells in the G0/G1, S, and G2/M phases from left to right, respectively. J) Represents histogram after 48 h treatment of M-CSF (50 ng/ml) used as positive control for proliferation. K) Showing quantification of microglial cells in each phase of cell cycle after respective treatments. Data are presented in percentage of cells in each phase. Results are expressed as means ± SEM of three independent experiments. Statistical analyses were carried out by using one-way ANOVA with post hoc Student-Newman-Keuls test (multiple comparisons). ^*^ *p* < 0.05; compared with percentage of control cells in each phase. (TIF 536 kb) Additional file 2: Figure S2. RBE did not significantly alter the protein levels of COX-2 in non-stimulated microglia. Cells were treated with RBE (50–300 μg/ml) for 24 h followed by lyses and protein estimation. During stimulation of microglia, one well of the 6-well plate was incubated with LPS (10 ng/ml) for 24 h to be used as positive control to validate the functionality of COX-2-specific antibody. Whole cell lysates were subjected to western blot for COX-2 and β-actin. Representative blots for COX-2 and β-actin are shown (upper panel) and densitometry analyses were performed (lower panel). To confirm equal sample loading, membranes were stripped and re-probed for β-actin and the data were used for normalization. Statistical analyses were carried out by using one-way ANOVA with post hoc Student-Newman-Keuls test (multiple comparisons). Results are expressed as means ± SEM of three independent experiments. ^***^ *p* < 0.001 compared with control cells. (TIF 323 kb) Additional file 3: Figure S3. (A-F). Possible influence of RBE alone on the expression of various genes. Cells were treated with RBE (50–300 μg/ml) for 24 h. LPS (10 ng/ml) was used in one of the wells as positive control to validate the functionality of primers and PCR protocol. Afterwards, gene expression of A) COX-2, B) mPGES-1, C) TNF-α, D) IL-1β, E) IL-6, and F) IL-10 was analyzed by real-time quantitative PCR. GAPDH was used as an internal control for normalization, and data were quantified by using comparative cycle threshold Ct method. Data are presented as percentage control of LPS. Results are expressed as means ± SEM of three independent experiments. Statistical analyses were carried out by using one-way ANOVA with post hoc Student-Newman-Keuls test (multiple comparisons). ^***^ *p* < 0.001 compared with LPS (10 ng/ml)-activated cells. (TIF 585 kb) Additional file 4: Figure S4. (A-C). Effects of RBE on the phosphorylation of p38MAPK, ERK, and JNK in non-activated microglia. Cells were treated with RBE (50–300 μg/ml) for 24 h followed by cell lyses and protein estimation. During stimulation, one of the wells in 6-well plate was incubated with LPS (10 ng/ml) for 30 min to be used as positive control to validate the functionality of antibodies against activated state of kinases. Whole cell lysates were subjected to western blots analyses. Representative blots (upper panel) and densitometry analyses (lower panel) are shown: A) p38 MAPK, B) pERK, and C) pJNK. Statistical analyses were carried out by using one-way ANOVA with post hoc Student-Newman-Keuls test (multiple comparisons). Results are expressed as means ± SEM of three independent experiments. **p* < 0.05; ***p* < 0.01; ****p* < 0.001 compared control cells. (TIF 963 kb) Additional file 5: Figure S5. (A-D). Effects of α-tocopherol on the release of PGE_2_ and cytokines. Influence of α-tocopherol on the release of PGE_2_, TNF-α, IL-1β, and IL-6 in LPS-activated microglia was also examined. Cells were pre-treated with RBE (50–300 μg/ml); subsequently, LPS (10 ng/ml) was added for 24 h. Afterwards, release of A) PGE_2_, B) TNF-α, C) IL-1β, and D) IL-6, was analyzed by using immunoassays. Data are presented as percentage control of LPS. Results are expressed as means ± SEM of three to four independent experiments. Statistical analyses were carried out by using one-way ANOVA with post hoc Student-Newman-Keuls test (multiple comparisons). ^*^ *p* < 0.05; ^**^ *p* < 0.01; ^***^ *p* < 0.001 compared with LPS (10 ng/ml)-activated cells. (TIF 685 kb)
