# Supplementary figures and images for: Rice bran derivatives alleviate microglia activation: possible involvement of MAPK pathway
Source: J Neuroinflammation. 2016 Jun 14;13:148. doi: 10.1186/s12974-016-0615-6 (PMC4908728; doi:10.1186/s12974-016-0615-6)

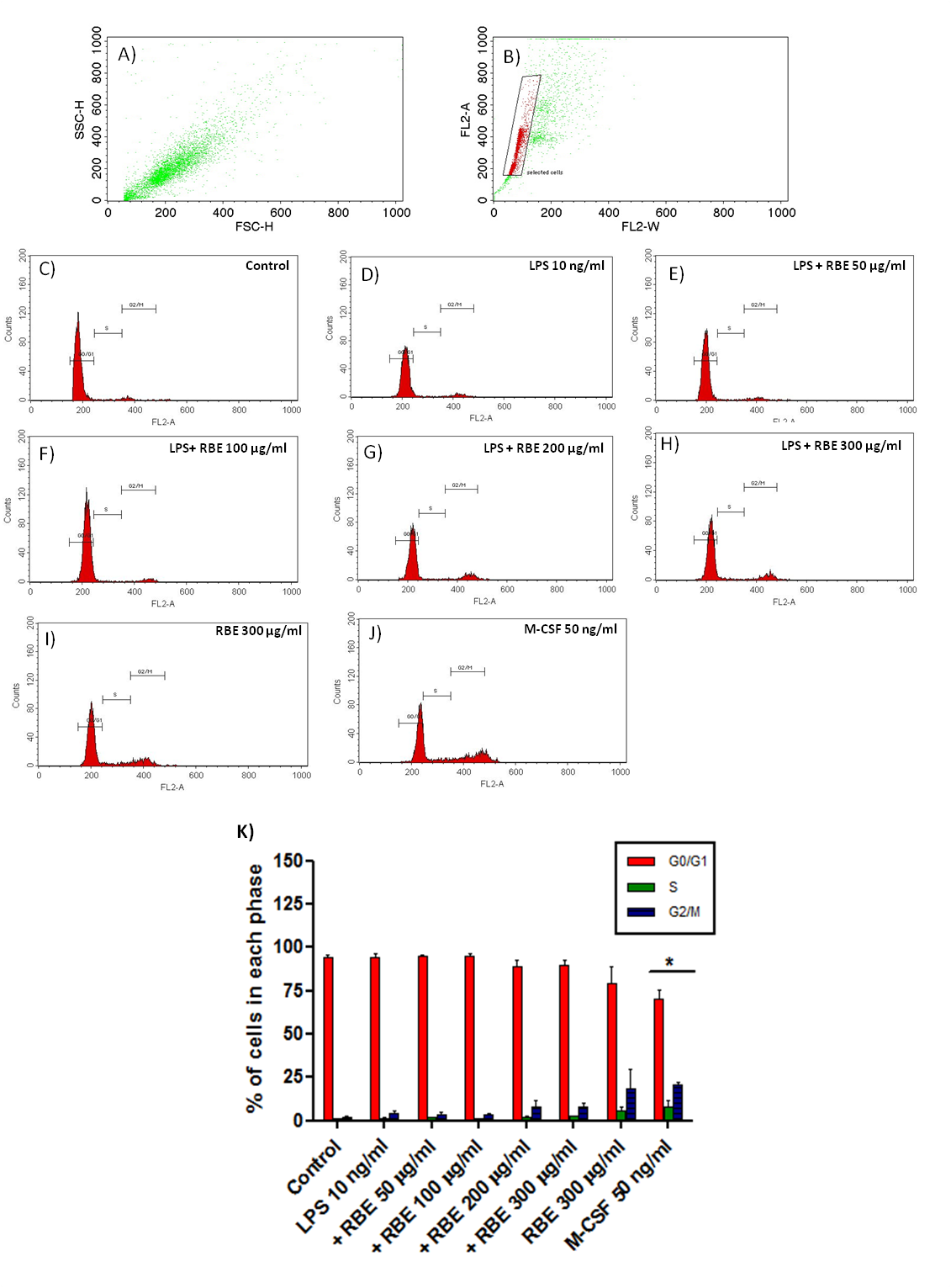

Supplement: Additional file 1: Figure S1. — (A-K). Possible effects of rice bran extract (RBE) on the proliferation of microglia. Microglia were treated with either RBE (300 μg/ml) alone or RBE (50–300 μg/ml) in combination with LPS (10 ng/ml) for a total of 48 h. Thereafter, samples were stained with propidium iodide and processed for proliferation assay (for detailed protocol, see the “Methods” section) by using flow cytometer. Samples were acquired with the FL-2 fluorescence channel set to a linear scale, in order to amplify the diploid DNA peak. Graph A) represents the dot plot of cells acquired on the basis of side scattered light (SSC) and forward scattered light (FSC) and B) represents the pulse processing by using pulse area vs. pulse width. Graphs C-I) are representative histograms after each treatment. Markers represent the percentage of cells in the G0/G1, S, and G2/M phases from left to right, respectively. J) Represents histogram after 48 h treatment of M-CSF (50 ng/ml) used as positive control for proliferation. K) Showing quantification of microglial cells in each phase of cell cycle after respective treatments. Data are presented in percentage of cells in each phase. Results are expressed as means ± SEM of three independent experiments. Statistical analyses were carried out by using one-way ANOVA with post hoc Student-Newman-Keuls test (multiple comparisons). * p < 0.05; compared with percentage of control cells in each phase. (TIF 536 kb) [file 12974_2016_615_MOESM1_ESM.tif]

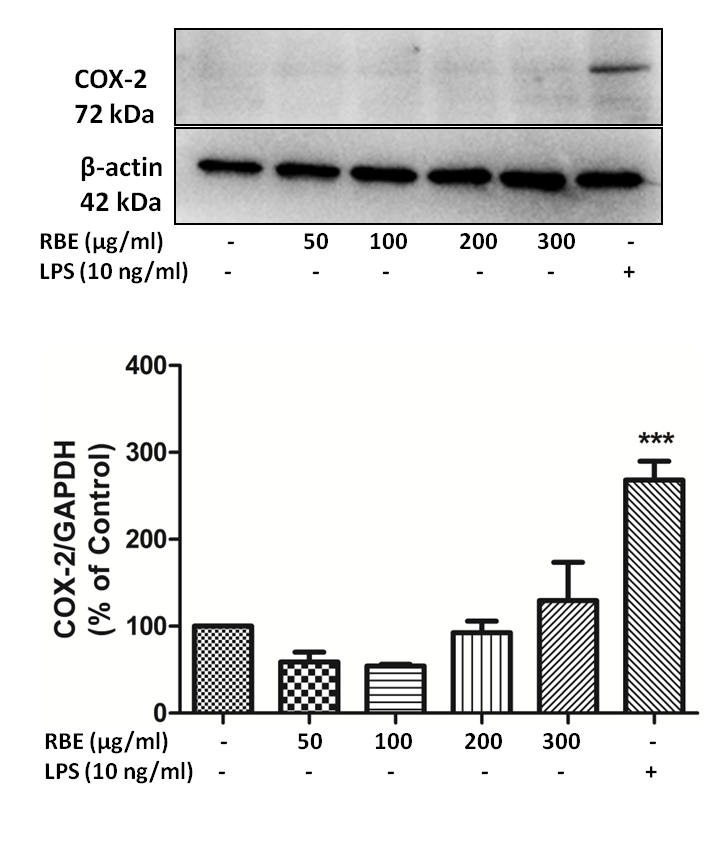

Supplement: Additional file 2: Figure S2. — RBE did not significantly alter the protein levels of COX-2 in non-stimulated microglia. Cells were treated with RBE (50–300 μg/ml) for 24 h followed by lyses and protein estimation. During stimulation of microglia, one well of the 6-well plate was incubated with LPS (10 ng/ml) for 24 h to be used as positive control to validate the functionality of COX-2-specific antibody. Whole cell lysates were subjected to western blot for COX-2 and β-actin. Representative blots for COX-2 and β-actin are shown (upper panel) and densitometry analyses were performed (lower panel). To confirm equal sample loading, membranes were stripped and re-probed for β-actin and the data were used for normalization. Statistical analyses were carried out by using one-way ANOVA with post hoc Student-Newman-Keuls test (multiple comparisons). Results are expressed as means ± SEM of three independent experiments. *** p < 0.001 compared with control cells. (TIF 323 kb) [file 12974_2016_615_MOESM2_ESM.tif]

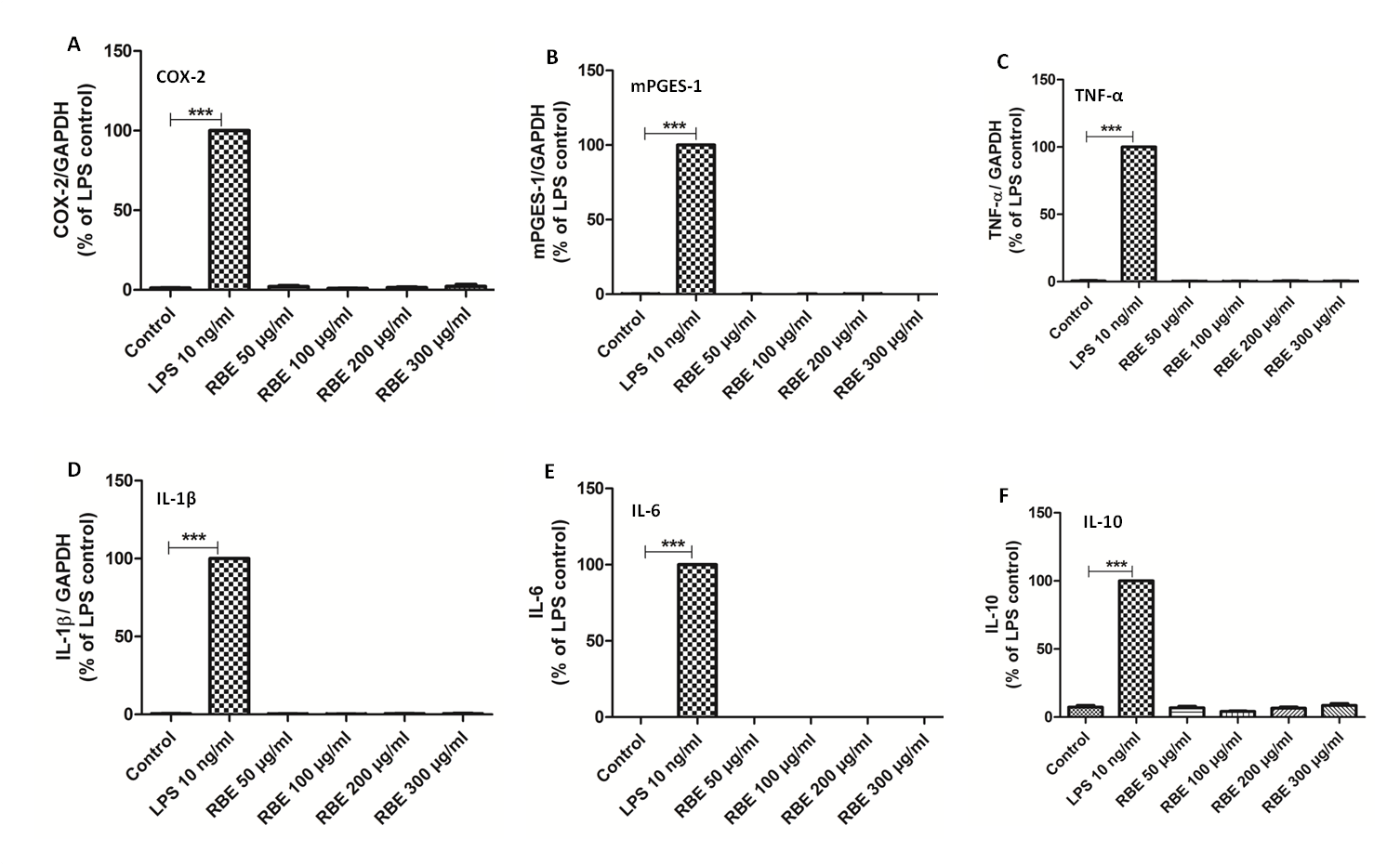

Supplement: Additional file 3: Figure S3. — (A-F). Possible influence of RBE alone on the expression of various genes. Cells were treated with RBE (50–300 μg/ml) for 24 h. LPS (10 ng/ml) was used in one of the wells as positive control to validate the functionality of primers and PCR protocol. Afterwards, gene expression of A) COX-2, B) mPGES-1, C) TNF-α, D) IL-1β, E) IL-6, and F) IL-10 was analyzed by real-time quantitative PCR. GAPDH was used as an internal control for normalization, and data were quantified by using comparative cycle threshold Ct method. Data are presented as percentage control of LPS. Results are expressed as means ± SEM of three independent experiments. Statistical analyses were carried out by using one-way ANOVA with post hoc Student-Newman-Keuls test (multiple comparisons). *** p < 0.001 compared with LPS (10 ng/ml)-activated cells. (TIF 585 kb) [file 12974_2016_615_MOESM3_ESM.tif]

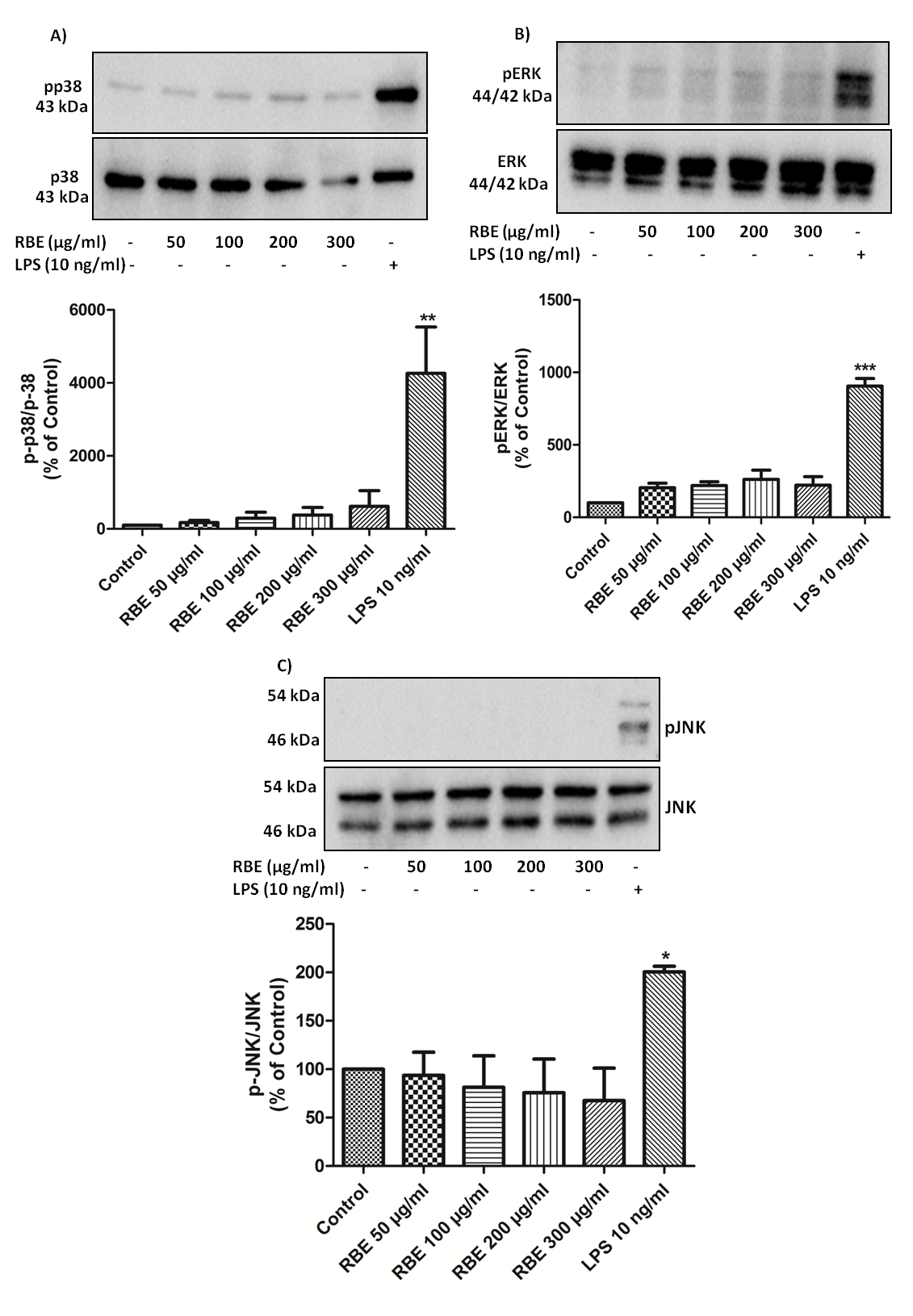

Supplement: Additional file 4: Figure S4. — (A-C). Effects of RBE on the phosphorylation of p38MAPK, ERK, and JNK in non-activated microglia. Cells were treated with RBE (50–300 μg/ml) for 24 h followed by cell lyses and protein estimation. During stimulation, one of the wells in 6-well plate was incubated with LPS (10 ng/ml) for 30 min to be used as positive control to validate the functionality of antibodies against activated state of kinases. Whole cell lysates were subjected to western blots analyses. Representative blots (upper panel) and densitometry analyses (lower panel) are shown: A) p38 MAPK, B) pERK, and C) pJNK. Statistical analyses were carried out by using one-way ANOVA with post hoc Student-Newman-Keuls test (multiple comparisons). Results are expressed as means ± SEM of three independent experiments. *p < 0.05; **p < 0.01; ***p < 0.001 compared control cells. (TIF 963 kb) [file 12974_2016_615_MOESM4_ESM.tif]

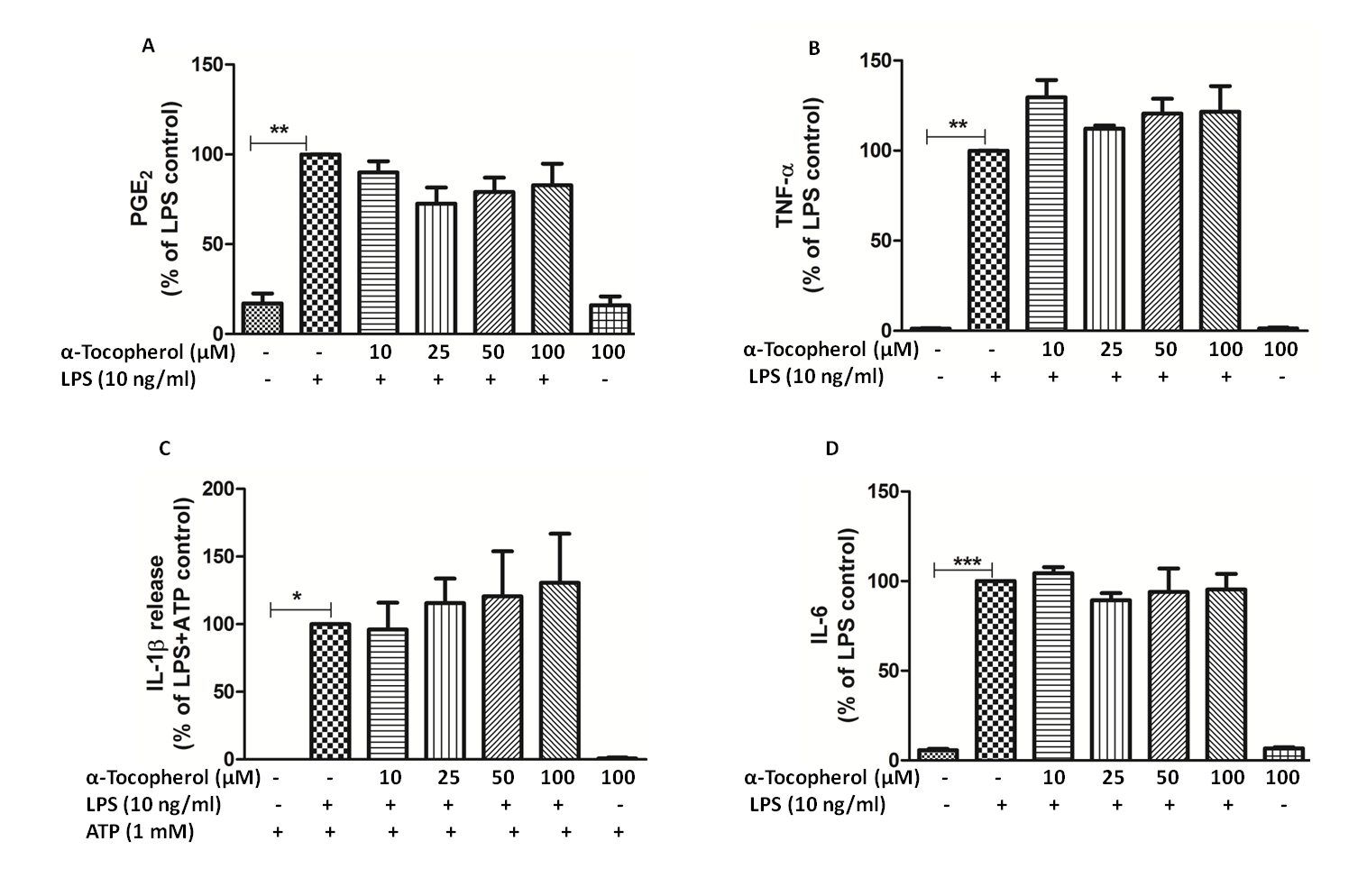

Supplement: Additional file 5: Figure S5. — (A-D). Effects of α-tocopherol on the release of PGE2 and cytokines. Influence of α-tocopherol on the release of PGE2, TNF-α, IL-1β, and IL-6 in LPS-activated microglia was also examined. Cells were pre-treated with RBE (50–300 μg/ml); subsequently, LPS (10 ng/ml) was added for 24 h. Afterwards, release of A) PGE2, B) TNF-α, C) IL-1β, and D) IL-6, was analyzed by using immunoassays. Data are presented as percentage control of LPS. Results are expressed as means ± SEM of three to four independent experiments. Statistical analyses were carried out by using one-way ANOVA with post hoc Student-Newman-Keuls test (multiple comparisons). * p < 0.05; ** p < 0.01; *** p < 0.001 compared with LPS (10 ng/ml)-activated cells. (TIF 685 kb) [file 12974_2016_615_MOESM5_ESM.tif]
